# Suicide in people prescribed opioid‐agonist therapy in Scotland, United Kingdom, 2011–2020: A national retrospective cohort study

**DOI:** 10.1111/add.16680

**Published:** 2024-10-22

**Authors:** Rosalyn Fraser, Alan Yeung, Megan Glancy, Matthew Hickman, Hayley E. Jones, Saket Priyadarshi, Kirsten Horsburgh, Sharon J. Hutchinson, Andrew McAuley

**Affiliations:** ^1^ School of Health and Life Sciences, Research Centre for Health (ReacH) Glasgow Caledonian University Cowcaddens Road Glasgow UK; ^2^ Public Health Scotland Gyle Square, South Gyle Crescent Edinburgh UK; ^3^ Bristol Medical School University of Bristol Tyndall Avenue Bristol UK; ^4^ Glasgow Alcohol and Drug Recovery Services Glasgow UK; ^5^ Scottish Drugs Forum Glasgow UK

**Keywords:** demography, epidemiology, injecting, modelling, opiates, opioid agonist treatment, Scotland, suicide

## Abstract

**Background and aims:**

Opioid dependence is associated with an increased risk of suicide. Drug‐related mortality among people with opioid dependence in Scotland has more than tripled since 2010; less is known about changes in suicide risk. We aimed to determine if opioid agonist therapy (OAT) in Scotland is protective against suicide and to measure trends in suicide rates in those with opioid dependence over time.

**Design:**

Retrospective cohort study.

**Setting:**

Scotland, UK.

**Participants:**

46 453 individuals in Scotland who received at least one prescription for OAT between 2011 and 2020 with over 304 000 person‐years (pys) of follow‐up.

**Measurements:**

We calculated standardised mortality ratios (SMR) using the age‐ and sex‐specific suicide rates in Scotland for years 2011–2020. We fitted multivariable competing‐risk regression models to estimate suicide rates by OAT exposure and to estimate trends over time, adjusting for potential confounders.

**Findings:**

There were 575 deaths classed as suicide among the cohort and the overall suicide rate was 1.89 (95% confidence interval [CI] = 1.74–2.05) per 1000 pys. Age and sex SMR for suicide was 7.05 times (95% CI = 6.50–7.65) higher than in the general population. After adjustment, OAT was shown to be highly protective against suicide, with rates more than three times greater (adjusted hazard ratio: 3.07; 95% CI = 2.60–3.62) off OAT compared with on OAT. Suicide rates decreased over time, falling from 2.57 (95% CI = 2.19–3.02) per 1000 pys in 2011–12 to 1.48 (95% CI = 1.21–1.82) in 2019–20.

**Conclusion:**

People with opioid dependence in Scotland appear to have a greater risk of suicide than the general population. Treatment is protective, with rates of suicide lower among those on opioid agonist therapy. Suicide rates have decreased over time, during a period in which drug‐related death rates in Scotland have risen to globally high levels.

## INTRODUCTION

Internationally, it is estimated that over 700 000 people take their own lives annually [1]. The global rate of suicide is ~9.4 deaths per 100 000 people, with higher rates among men (13.3 deaths per 100 000) than women (5.7 deaths per 100 000) [2]. In Scotland, suicide rates have fallen slightly from 17.6 deaths per 100 000 people (*n* = 899) in 2002 to 13.9 deaths per 100 000 people in 2022 (*n* = 762) [3]. In line with global evidence, rates are consistently higher among males than females.

People with a substance use disorder are at a greatly increased risk of suicide compared with the general population [4]. Although suicidal intent can be difficult to determine in many drug‐related deaths (DRDs), there is evidence that opioid dependence is associated with a substantial increased risk of suicide, with suicide risk found to be nearly seven times higher in those with opioid dependence [4].

Opioid‐agonist therapy (OAT) with methadone or buprenorphine is the standard care for opioid dependence and categorised as ‘essential medicines’ by the World Health Organization [5]. OAT is available free of charge in Scotland, and mainly delivered through community‐based settings, including drug treatment services. A large and consistent evidence base has shown that OAT reduces the risk of overall mortality and DRD in particular with up to 60% reduced risk [6, 7, 8, 9, 10]. A recent Scotland‐wide study showed that OAT was protective against DRD over time, with rates being almost 3.5 times higher for those off OAT compared to those on OAT, and an average 70% reduced risk of DRD overall for those on OAT [11]. Despite this, opioid‐related deaths increased 127% between 2011 and 2020 from 524 to 1192 with rates now among the highest recorded internationally [12].

There is emerging evidence that OAT is protective against suicide, with risk reduced by ~50% for those on OAT compared to those off OAT [6]. Although most studies lack power to detect any effect during initiation of OAT [13, 14, 15], one cohort study spanning 20 years did show a greater than three‐fold increase in risk of suicide during the first 2 weeks of receiving treatment [16]. Suicide risk is also strongly elevated in the immediate period after cessation of OAT, reported to be up to 17 times higher [13, 14]. Overall, the evidence base on suicide risk and the preventative role of OAT to date is severely limited in comparison to other mortality research among people who use drugs [6] with the most recent United Kingdom (UK) study impacted by low numbers of suicides across the study period [14].

In this study, we examined the rates of suicide among people prescribed OAT in Scotland. Our objectives were to determine whether OAT is protective against suicide and whether suicide rates in people with opioid dependence followed trends in DRD or general population suicide rates in Scotland.

## METHODOLOGY

### Study design and population

We undertook a national retrospective cohort study of individuals who received at least one prescription in Scotland for OAT between 1 January 2011 and 31 December 2020. Deterministic linkage was carried out at Public Health Scotland (PHS) between prescribing data, mortality data and other administrative healthcare data. Linkage was done using the Community Health Index (CHI) number, a unique identifier assigned to individuals accessing the National Health Service (NHS) in Scotland [17].

### Data sources

Further details on data sources are included within Data S1. In brief, prescription records were linked using the CHI number. CHI coverage per OAT prescription was 74% overall, but has improved over time (range: 64%–81%). To define the exposure, the date of prescription reimbursement was used to estimate periods on/off OAT (Data S1) [11].

For the outcome, mortality data that included date and cause of death were obtained from Scotland's death registry, maintained by National Records Scotland (NRS). The primary outcome measure for this study was suicide. NRS defines this as all deaths recorded as a suicide, whether of determined or undetermined intent (Data S1, Table 1).

**TABLE 1:** Characteristics of study cohort^a^.

	*N* (%)
Total	46 453
Sex	
Female	15 219 (32.8)
Male	31 234 (67.2)
Age group, y (at cohort‐entry)	
15–24	3026 (6.5)
25–34	18 093 (38.9)
35–44	18 095 (39.0)
45–54	6080 (13.1)
55–69	1159 (2.5)
SIMD quintile (last known) (missing = 208, 0.4%)	
1 (most deprived)	24 426 (52.6)
2	11 691 (25.2)
3	5650 (12.2)
4	3050 (6.6)
5 (least deprived)	1428 (3.1)
Self‐harm hospital admission (ever)	
No	35 293 (76.0)
Yes	11 160 (24.0)
Charlson Comorbidity Index (ever)	
0 (none)	30 619 (65.9)
1–2 (medium)	10 619 (22.9)
3+ (high)	5215 (11.2)
Deaths	
All‐cause	6947 (15.0)
Drug‐related	4076 (8.8)
Suicides (including undetermined intent)	575 (1.2)
Suicides (excluding undetermined intent)	334 (0.7)
Suicides (drug‐related including undetermined intent)	263 (0.6)
Suicides (drug‐related excluding undetermined intent)	62 (0.1)

Abbreviation: SIMD, Scottish Index of Multiple Deprivation.

^a^ A fuller table of cohort characteristics is available in both Data S3 and McAuley *et al*. [11].

PHS‐held data was accessed to define a number of confounder variables that were informed by existing evidence on suicide risk and OAT (Data S1, Table 1): hospital admissions for self‐harm and mental health from SMR01 (national record of inpatient and day case hospital episodes) and SMR04 (national record of mental health inpatient and day case hospital episodes); prescriber type information (whether prescribed by a general practitioner [GP] or other prescriber) for prescriptions contained within the national Prescribing Information System (PIS); deprivation status from the Scottish Index of Multiple Deprivation (SIMD), approximated from an individual's last known postcode of residence, obtained from the CHI database held by PHS; and the Charlson Comorbidity Index (CCI), based on hospital admissions obtained from SMR01 [18].

### Exclusion criteria

We excluded patients known to have died before 1 January 2011 (*n* = 25); those age <15 or >69 years at baseline prescription (*n* = 304); anyone not resident in Scotland (*n* = 15); and individuals whose date of prescription reimbursement was more than 60 days after their date of death, such that we were unable to specify their date of last treatment (*n* = 163) (Data S2).

### Statistical analysis

Suicide rates were calculated as the rate per 1000 person‐years (pys), over a 10‐year analysis period (1 January 2011–31 December 2020). Follow‐up commenced on 1 January 2011 for those who received an OAT prescription in 2010 and, for all other people, on the estimated date of their first prescription. Follow‐up ended at the earliest of date of death from any cause, 24 months after cessation of treatment episode or 31 December 2020 (i.e. end of study period). Censoring at 24 months was implemented to minimise potential misclassification of the pys at risk among those with a higher likelihood of having ceased drug use [11].

We first followed other studies [6, 13, 14] in estimating crude suicide mortality rates (CSMRs) stratified by key demographics and comorbidities: OAT exposure at time of death (on or off); attained age‐group; sex; SIMD (numerical, quintiles 1–5); prescriber type (GP or other); previous hospital admission for self‐harm (yes or no); and CCI (none, [0]; medium, [1–2]; high [3+]). Both OAT exposure and age‐group are time‐varying covariates. All other covariates were measured at baseline. We, then, calculated age and sex standardised mortality ratios (SMR) using the age and sex specific suicide rates in Scotland for years 2011 to 2020. We further stratified SMRs by bi‐yearly period and compared our SMR results to those from similar studies [13, 14].

An attributable risk fraction (ARF) was calculated using Levin's formula [19] (ARF = P1(RR‐1)/1 + P1(RR‐1)) where P1 was the estimated prevalence of opioid dependence in the Scottish population [20], and RR was our age and sex adjusted SMR for suicide. The ARF is an estimate of the proportion of the health problem (e.g. suicides) that has occurred in the population because of the risk factor (opioid dependence) [19].

We examined the association between suicide and OAT by plotting CSMRs over time, stratified by OAT status. We also further stratified CSMRs over time for males and females, by age‐group and by OAT status.

To account for high drug‐related and all‐cause mortality (ACM) among the cohort over the study period [11], we fitted competing‐risks regression models using the Fine and Gray method [21]. This accounts for the presence of other events that preclude the event of interest (suicide). In this case, the competing event was ACM (excluding suicides). The Fine and Gray approach models the hazard of suicide (the ‘subdistribution hazard’), accounting for the presence of ACM, under an assumption of proportional subdistribution hazards. We fitted univariable competing‐risks regression models to estimate crude associations between covariates and suicide, and multivariable models to estimate if OAT is protective and to estimate trends over time, adjusting for likely confounders. Likelihood ratio tests, with and without a frailty term included, indicated no evidence of overdispersion in our models.

### Sensitivity analysis

We conducted a series of sensitivity analyses to test various assumptions in our analysis plan. First, we removed suicides recorded as undetermined intent from our outcome measure, as these may have been misclassified as suicides. Second, we removed censoring of follow‐up time to compare model estimates to those in the primary analysis, where we adjusted for survival bias. Third we ended follow‐up time at 29 February 2020 to restrict analysis to periods not including the coronavirus disease 2019 (COVID‐19) pandemic. Fourth, we fitted a competing risks regression model with only DRD (excluding suicides) as the competing event.

Analysis was conducted using R (v4.1.2) and Stata (v13).

The analysis plan was not pre‐registered, and results should be considered exploratory.

### Role of the funding source

This study was funded by the Scottish Government Drug Death Task Force. The funding source was independent of the design of this study and did not have any role during its execution, analyses, data interpretation, writing or decision to submit results. This study was also supported by PHS and the National Institute for Health and Care Research (NIHR). NIHR funding was through the Programme Grants for Applied Research programme (grant reference number, RP‐PG‐0616‐20 008) and Health Protection Research Unit in Behavioural Science and Evaluation (HPRU BSE). The views expressed are those of the author(s) and not necessarily those of the NIHR or the Department of Health and Social Care. All authors were not precluded from accessing data in the study and take responsibility for the integrity of the data and accuracy of analysis.

## RESULTS

### Patient cohort characteristics

The cohort consisted of 46 453 individuals who received at least one CHI‐identified prescription of methadone, buprenorphine or Suboxone between 1 January 2011 and 31 December 2020, with around 304 000 pys of follow‐up (Data S2). Two thirds (67%) were male, almost half (45%) were under 35‐years old at beginning of follow‐up, and over half (53%) were categorised as living in the most deprived quintile (quintile 1) in Scotland (Table 1). Further details on cohort characteristics are available in Data S3 [11].

### Overall rates

There were 575 deaths classified as suicide among the cohort within the main analysis, accounting for 1.2% of the sample overall and 8.3% of all deaths (after censoring of follow‐up) recorded (Table 1). The overall suicide rate in the cohort was 1.89 (95% CI = 1.74–2.05) per 1000 pys (Table 2).

**TABLE 2:** Crude suicide mortality rates, hazard ratios and adjusted hazard ratios for suicides among people prescribed OAT in Scotland 2011 to 2020.

Variable	Deaths	PY	Mortality/1000 PY (95% CI)	HR^a^	*P*‐value	Adj. HR^a^	*P*‐value
Total	575	304 043	1.89 (1.74–2.05)				
OAT exposure							
On	256	223 973	1.14 (1.01–1.29)	1 (ref)		1 (ref)	
Off	319	80 070	3.98 (3.57–4.45)	3.04 (2.58, 3.58)	<0.001	3.07 (2.60, 3.62)	<0.001
Sex							
Male	415	203 776	2.04 (1.85–2.24)	1 (ref)		1 (ref)	
Female	160	100 267	1.60 (1.37–1.86)	0.79 (0.66, 0.95)	0.012	0.79 (0.65, 0.95)	0.013
Age, y							
Under 35	148	81 989	1.81 (1.54–2.12)	0.86 (0.70, 1.06)	0.161	0.79 (0.64, 0.97)	0.028
35–44	265	139 043	1.91 (1.69–2.15)	1 (ref)		1 (ref)	
45–54	162	83 011	1.95 (1.67–2.28)	1.03 (0.84, 1.25)	0.781	1.13 (0.92, 1.38)	0.252
Time period							
2011–2012	149	57 890	2.57 (2.19–3.02)	1 (ref)		1 (ref)	
2013–2014	123	60 670	2.03 (1.70–2.42)	0.80 (0.58, 1.09)	0.153	0.77 (0.58, 1.03)	0.081
2015–2016	99	61 720	1.60 (1.32–1.95)	0.71 (0.51, 0.98)	0.036	0.64 (0.48, 0.87)	0.004
2017–2018	113	62 310	1.81 (1.51–2.18)	0.82 (0.60, 1.12)	0.210	0.74 (0.55, 1.00)	0.050
2019–2020	91	61 460	1.48 (1.21–1.82)	0.72 (0.52, 0.99)	0.040	0.62 (0.46, 0.84)	0.002
SIMD (deprivation index)							
SIMD 3, 4, 5 (least deprived)	105	60 522	1.73 (1.43–2.10)	1 (ref)		1 (ref)	
SIMD 2	157	76 014	2.07 (1.77–2.42)	1.21 (0.94, 1.55)	0.133	1.24 (0.97, 1.60)	0.087
SIMD 1 (most deprived)	313	167 507	1.87 (1.67–2.09)	1.09 (0.88, 1.37)	0.422	1.16 (0.93, 1.45)	0.196
Mental health disorder admission							
No	458	274 146	1.67 (1.52–1.83)	1 (ref)		1 (ref)	
Yes	117	29 897	3.91 (3.26–4.69)	2.26 (1.85, 2.77)	<0.001	2.03 (1.62, 2.53)	<0.001
Self‐harm hospital admission							
No	375	234 132	1.60 (1.45–1.77)	1 (ref)		1 (ref)	
Yes	200	69 911	2.86 (2.49–3.29)	1.71 (1.44, 2.03)	<0.001	1.57 (1.30, 1.89)	<0.001
Prescriber type							
General practitioner	364	194 621	1.87 (1.69–2.07)	1 (ref)		1 (ref)	
Other	211	109 422	1.93 (1.68–2.21)	1.02 (0.86, 1.20)	0.858	0.92 (0.77, 1.09)	0.324
Charlson Comorbidity Index							
0 (none)	409	195 481	2.09 (1.90–2.31)	1 (ref)		1 (ref)	
1–2 (medium)	115	74 173	1.55 (1.29–1.86)	0.73 (0.60, 0.90)	0.003	0.65 (0.53, 0.81)	<0.001
3+ (high)	51	34 388	1.48 (1.13–1.95)	0.63 (0.47, 0.85)	0.002	0.50 (0.37, 0.68)	<0.001

Abbreviations: Adj., adjusted; HR, hazard ratio; OAT, opioid agonist therapy; PY, person years; SIMD, Scottish Index of Multiple Deprivation.

^a^ Modelled using competing risks regression, with all‐cause mortality (excluding suicides) as the competing event.

### Effect of OAT

The CSMR for those off OAT was more than three times higher relative to those on OAT, with rates of 3.98 (95% CI = 3.57–4.45) and 1.14 (95% CI = 1.01–1.29) per 1000 pys, respectively (Table 2). This protective effect of OAT was maintained after adjustment in the multivariable model where the suicide rate off OAT was more than three times greater (adjusted hazard ratio [aHR] = 3.07; 95% CI = 2.60–3.62) compared to on OAT.

### Trends over time

**FIGURE 1:**
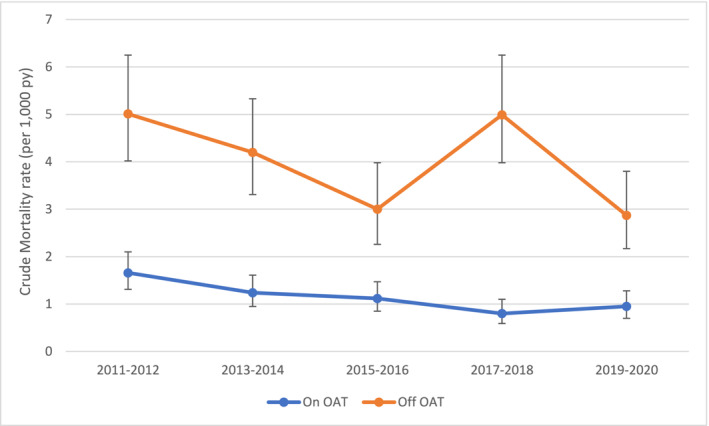
Crude suicide mortality rates over time, by opioid agonist therapy (OAT) exposure.

There was a decrease in suicide rates over time, with CSMRs falling from 2.57 (95% CI = 2.19–3.02) per 1000 pys in 2011 to 2012, to 1.48 (95% CI = 1.21–1.82) in 2019 to 2020. Consistently lower suicide rates were observed among those on OAT across the time series (Figure 1), but with a reduction in rates in both groups over time. The only exception to these trends was a spike in 2017 to 2018 among those off OAT (Data S5). In the adjusted model, suicide rates were significantly reduced across the periods of 2015 to 2016, 2017 to 2018 and 2019 to 2020 compared to 2011 to 2012.

### Comparison to the general population

Age and sex SMR was 7.05 (95% CI = 6.50–7.65) (Table 3). When stratified by time‐period, the SMR also decreased over time, falling from 9.77 (95% CI = 8.32–11.47) in 2011 to 2012, to 5.50 (95% CI = 4.48–6.75) in 2019 to 2020. The ARF for the proportion of suicides that were estimated to be because of opioid dependence in Scotland, was calculated as 16.0% (95% CI = 14.5%–17.6%) (Data S7).

**TABLE 3:** Age and sex SMRs, for suicides among people prescribed OAT in Scotland, 2011–2020: overall and by time period.

Time period	Observed no. suicides	Expected no. suicides	SMRs (95% CI)^a^
Overall	575	81.55	7.05 (6.50–7.65)
2011–2012	149	15.26	9.77 (8.32–11.47)
2013–2014	123	16.19	7.60 (6.37–9.07)
2015–2016	99	16.66	5.94 (4.88–7.23)
2017–2018	113	16.89	6.69 (5.57–8.05)
2019–2020	91	16.55	5.50 (4.48–6.75)

Abbreviations: OAT, opioid agonist therapy; SMRs, standardised mortality ratios.

^a^ Calculation breakdown for the SMR is shown in Table 1, Data S6.

### Differences by sex, age group and other factors

Suicide rates were slightly lower among females than males, with CSMRs of 1.60 (95% CI = 1.37–1.86) and 2.04 (95% CI = 1.85–2.24) per 1000 pys, respectively (Table 2). There were minimal differences in the CSMRs across age groups. In the multivariable model, females remained at significantly lower rate of suicide compared to males (aHR = 0.79; 95% CI = 0.65–0.95) and those age under 35 had a reduced rate of suicide compared to those age 35 to 44 (aHR = 0.79; 95% CI = 0.64–0.97).

Other covariates were associated with an increased rate of suicide after adjustment in the multivariable model, including having had a previous hospital admission for a mental health disorder (aHR = 2.03; 95% CI = 1.62–2.53) and having a previous self‐harm hospital admission (aHR = 1.57; 95% CI = 1.30–1.89). Comorbidity was associated with a reduced rate of suicide (CCI 1–2: aHR = 0.65; 95% CI = 0.53–0.81), with potential evidence of cumulative effect (CCI 3+: aHR = 0.50; 95% CI = 0.37–0.68), compared to having no history of comorbidity.

### Sensitivity analyses

After removing deaths of undetermined intent (*n* = 241), evidence for the protective effect of OAT held (aHR = 3.14; 95% CI = 2.52–3.90) and results were generally similar to those from the main model but with wider confidence intervals because of reduced statistical power (Data S4, Table 1). However, in contrast to the main model, there was evidence for a higher rate of suicide among people who fall into the most deprived group (SIMD 1) (aHR = 1.45; 95% CI = 1.07–1.96), compared to those in SIMD groups 3 to 5 (least deprived). Removing censoring of follow‐up completely for those off OAT also gave overall similar results to the main model (Data S4, Table 2), but with a reduced estimate of the protective effect of OAT (aHR = 2.57; 95% CI = 2.18–3.02). This is as we assumed a priori because of those who joined the cohort earlier accumulating more pys over time than those joining later, therefore, confirming the need to censor follow‐up time for those off OAT when examining trends for consistency. Restricting follow‐up time to end on the 29 of February 2020 (i.e. omitting COVID‐19 era data), made minimal difference to results in the main model (Data S4, Table 3).

When modelling with DRD (excluding suicides) as the competing risk (Data S4, Table 4), we still observed the increased rate off OAT (aHR = 3.24; 95% CI = 2.74–3.83) and results were equivalent to the main model.

## DISCUSSION

### Main findings

OAT was associated with a substantial and sustained reduction in suicide rate in Scotland between 2011 and 2020, with a more than two‐thirds reduction in rates in people with opioid dependence on OAT compared to when off OAT. There was evidence of a decline in suicide rates over a period where the rate of DRDs in the same population more than trebled to globally high levels. Suicide rates in people with opioid dependence were still eight times higher than the rate in Scotland's general population with an estimated attributable risk fraction of 16%.

### Comparison to other studies

To our knowledge, this is one of the largest cohort studies internationally to have examined the rate of suicide in those prescribed OAT to date. The only other UK study [14] to date found preliminary evidence of an association between suicide and OAT exposure because of the small number of suicides in their cohort (*n* = 46), less than 10% of the number we observed within this study.

The overall suicide rate of 1.89 per 1000 pys we observed is higher than recorded in Australian (New South Wales [NSW]) and English studies of OAT recipients covering broadly similar periods, which reported suicide rates of 1.0 per 1000 pys and 1.15 per 1000 pys, respectively [13, 14]. Moreover, our SMR of 7.05 per 1000 pys was higher than recorded in NSW (5.6 per 1000 pys), but similar to England (7.5 per 1000 pys). An Italian longitudinal study also found very high rates of suicide in their OAT population compared to the general population, with an SMR of 6.4 per 1000 pys [22].

Treatment delivery models for OAT may differ between Scotland and other countries and, therefore, impact on OAT effectiveness. In Scotland, OAT is mainly delivered in community settings such as primary care, drug treatment and community pharmacy services via the NHS at no cost to the patient. In England, OAT is also free at point of delivery and prescriptions can be received from primary care via the NHS. However, treatment is also commonly provided by licensed Non‐Governmental Organisations who account for ~30% of those on OAT in England [23]. In NSW, historically there has been a mix of public and privately funded treatment. From July 2023, OAT medicines in Australia became part of the Section 100 Highly Specialised Drugs Program and a new OAT Community Pharmacy Program was established that provides nationally consistent payment arrangements for OAT services delivered through community pharmacies, aiming to address patient equity and affordability issues [24]. Before the recent arrangements, patients paid significant out of pocket costs, which were often a meaningful barrier to treatment access or continuation.

We reported a strong protective effect of OAT on suicide, which confirms and further strengthens prior research in this area [6]. The suicide rate for those off OAT in our study was, however, 3.98 per 1000 pys, much higher than a previous global systematic review that reported an off OAT suicide rate of 1.36 per 1000 pys [6]. The benefit of OAT exposure in reducing suicide rates is likely attributable to a combination of factors, including OAT facilitating access to other support services. Additionally, because OAT stabilises drug use and overall lifestyle, individuals could have improved quality of life and reduced psychological distress [25]. Engaging with OAT also provides opportunities to build therapeutic relationships and reduce isolation, a well‐established risk factor for suicide [26].

We found evidence of suicide rates among people with opioid use disorder decreasing over time, in contrast to other main causes of mortality within this cohort in Scotland. DRD risk in those prescribed OAT in Scotland more than trebled between 2011 and 2012 and 2019 and 2020 and has been declared a ‘public health emergency’ by the Scottish Parliament [11]. The decline in suicide rates in this OAT cohort mirrors falls in overall suicide rates in Scotland over the same period [3]. Population‐level suicide rates in Scotland had been declining since the millennium, but have stalled since 2016 [27].

Despite reductions, preventing suicide, particularly in this OAT cohort, should still be a key public health priority, as shown by a high ARF of 16% despite prevalence of opioid dependence of <1.5% [20]. Additional suicide prevention in the community (including increased engagement with OAT) is needed for this population, targeted to those at most risk. This includes people with opioid dependence with a self‐harm or a mental illness history, who are especially vulnerable as this study and previous work has shown [13, 14]. Examples of current suicide prevention support available in Scotland include provision of Applied Suicide Intervention Skills Training (ASIST) [28] and STORM [29] Skills Training for staff working within drug treatment services.

### Limitations

Our study has several limitations. We have previously considered limitations with prescribing data and the classification of people on and off OAT in detail in our work on DRD risk among the same cohort [11]. Principally, prescribing dates were missing for the majority of prescriptions, so an algorithm was generated to estimate OAT start and end dates. This meant that we were unable to determine at this time differences in suicide rates in the first 4 weeks after initiation of OAT and in the first 4 weeks after stopping OAT, which are both documented high‐risk periods [6, 13, 14, 16]. It is plausible that patients who stop taking OAT have key differences to those who do not. For example, patients may stop OAT because of poor mental health and withdrawal from society, both high risk factors for suicide. Therefore, we acknowledge the possibility of measurement error in this study, and that our findings are associations and not causal.

We did not explore differences between buprenorphine and methadone as most OAT prescriptions in the study period were for methadone. However, future work may allow for this comparison with methadone share of OAT prescribing in Scotland declining in recent years [30]. A comparable study in England found no difference in the adjusted rates of suicide between buprenorphine and methadone when comparing time on and off OAT [14].

A further limitation is in the potential misclassification of suicides in the absence of clear evidence regarding intentionality. NRS classifies both deaths of known intent and those of undetermined intent as a ‘probable suicide’ [3]. To account for this possible uncertainty, in sensitivity analysis we removed those deaths of undetermined intent, but observed broadly similar results suggesting misclassification bias to be minimal.

Changes to OAT prescribing in Scotland took place from March 2020 because of the COVID‐19 pandemic, with less daily dispensing and more take‐home dosing [31]. We, therefore, conducted a further sensitivity analysis, which ended follow‐up time at the end of February 2020 (immediately before the first diagnosis of COVID‐19 in Scotland). This change also made little difference to our results. Concerns about potential rises in suicide during the pandemic related to enforced isolation do not appear to have manifested at population level [32]. However, analysing the impact of COVID‐19 on mortality among people who use drugs, including suicides, should include the whole pandemic period and its aftermath to determine any immediate and delayed impacts [32].

We acknowledge the limitations of using internal time‐varying covariates in a Fine‐Gray subdistribution hazards model. Because of the nature of the subdistribution hazard set, defining these covariates can be difficult for subjects who have experienced a competing event, and we lose the ability to estimate the effect of these covariates on the cumulative incidence function [33]. Therefore, we have refrained from interpreting the results in terms of risk, because of its distinct probabilistic interpretation, and have instead used terms such as rates and hazard ratios.

Finally, although we included covariates representing a number of possible confounders in our modelling, some had limitations and others were missing. For example, covariates on mental health and self‐harm are likely to miss people diagnosed or exposed without admissions to hospital, so effect sizes are likely to be underestimated and not reflect the true scale for prevention through better diagnosis and targeting interventions to people with mental health and self‐harm history. Further, we did not have information on other covariates known to be associated with suicide such as homelessness and prison, therefore, the potential for residual confounding cannot be discounted. We, therefore, acknowledge that if confounding variables used in the calculation of the SMR and ARF are not well adjusted for, the ARF value may represent an overestimation.

## CONCLUSION

People with opioid dependence in Scotland are at substantially greater risk of suicide than the general population. However, during a period where DRD rates in Scotland rose to globally high levels, our study suggests that suicide rates in the same at‐risk population may have declined and followed trends in suicide in the community. There is strong evidence that rates of suicide are lower among those on OAT highlighting the importance of engaging people with opioid dependence in drug treatment to reduce risk.

## AUTHOR CONTRIBUTIONS

**Rosalyn Fraser**: Analysis and interpretation of data; drafting the article; final approval to submit the manuscript. **Alan Yeung**: Acquisition of the data; analysis and interpretation of the data; revision of the article; approval to submit the manuscript. **Megan Glancy**: Analysis and interpretation of the data; revision of the article; approval to submit the manuscript. **Matthew Hickman**: conceptualization and design of the study; interpretation of the data; revision of the article; approval to submit the manuscript. **Hayley E. Jones**: **C**onceptualization and design of the study; revision of the article; approval to submit the manuscript. **Saket Priyadarshi**: Revision of the article; approval to submit the manuscript. **Kirsten Horsburgh**: Revision of the article; approval to submit the manuscript. **Sharon Hutchinson**: conceptualization and design of the study; acquisition of the data; interpretation of the data; revision of the article; approval to submit the manuscript. **Andrew McAuley**: conceptualization and design of the study; interpretation of the data; revision of the article; approval to submit the manuscript.

## DECLARATION OF INTERESTS

We declare no competing interests.

## Supporting information


**Data S1–S7.** Supplementary Material.

## Data Availability

This study used pseudonymised patient‐level data from Public Health Scotland. To protect patient confidentiality, we cannot publish patient level data. Other researchers can use patient‐level data by Public Health Scotland in a secure environment by applying to the electronic Data Research and Innovation Service (eDRIS). Details of the application process and conditions of access are provided by eDRIS at https://www.isdscotland.org/Products-and-Services/eDRIS/.
