# Supplementary material for: Suicide in people prescribed opioid‐agonist therapy in Scotland, United Kingdom, 2011–2020: A national retrospective cohort study
Source: Addiction. 2024 Oct 22;120(2):276–84. doi: 10.1111/add.16680 (PMC11707309; doi:10.1111/add.16680)
Supplement: Supplementary file 1 — Data S1–S7. Supplementary Material. [file ADD-120-276-s001.docx]

**Supplementary Files**

**Contents**

[Supplementary File S1 1](#_Toc159412083)

[DATA SOURCES 1](#_Toc159412084)

[Exposure: Opioid agonist treatment (OAT) 1](#_Toc159412085)

[Outcome: Mortality data 1](#_Toc159412086)

[Supplementary File S2 3](#_Toc159412087)

[INCLUSION AND EXCLUSION CRITERIA 3](#_Toc159412088)

[Supplementary File S3 4](#_Toc159412089)

[FURTHER DETAILS ON COHORT CHARACTERISTICS 4](#_Toc159412090)

[Supplementary File S4 6](#_Toc159412091)

[SENSIVITY ANALYSIS 6](#_Toc159412092)

[(i) EXCLUDING SUICIDES OF UNDETERMINED INTENT 6](#_Toc159412093)

[(ii) REMOVING CENSORING OF FOLLOW UP TIME 8](#_Toc159412094)

[(iii) COVID-19 CENSORING 10](#_Toc159412095)

[(iv) DRUG RELATED DEATHS (EXCLUDING SUICIDES) AS THE COMPETING EVENT 12](#_Toc159412096)

[Supplementary File S5 14](#_Toc159412097)

[ASSOCIATIONS BETWEEN SUICIDE, OAT, SEX AND AGE GROUP 14](#_Toc159412098)

[Supplementary File S6 16](#_Toc159412099)

[STANDARDISED MORTALITY RATIO (SMR) BREAKDOWN AND COMPARISON 16](#_Toc159412100)

[Supplementary File S7 19](#_Toc159412101)

[ATTRIBUTABLE RISK FRACTION (ARF) CALCULATION 19](#_Toc159412102)

#

# **Supplementary File S1**

## **DATA SOURCES**

### **Exposure: Opioid agonist treatment (OAT)**

**The following information on the exposure variable was first reported in McAuley et al 2023.^1^**

From Scotland’s national Prescribing Information System (PIS) we obtained all prescriptions for methadone, buprenorphine and buprenorphine-naloxone, between 2010 and 2020. For each prescription record, we obtained the reimbursement date, prescription date (where available), health board of residence and type of medication.

Reimbursement dates are available for all prescriptions and are aggregated by month, with the date always falling at the end of the month. Payments are made when the prescription is fully dispensed, which creates a time-lag within the data: for example, a 28-day prescription made in mid-March may not be fully dispensed until mid-April and the date of reimbursement will therefore fall on 30 April. Prescription date is available only for a subset of records from electronic general practitioner prescriptions. In our sample, 43% of all records had a prescription date and the proportion of records with a prescription date has decreased over time from 57% in 2010 to 33% in 2020. Moreover, very few individuals overall (4.5%) had a complete record of prescription dates throughout the study period. We therefore used the date of reimbursement to determine periods on/off OAT for consistency.

For the 43% of prescriptions with a prescription date, the average (mean) number of days between date of prescription and reimbursement was 40 (SD, 20), consistent with previous analysis.^3^ For the subset of our sample with a complete record of prescription dates, the median number of days between consecutive prescription dates was 28 (IQR, 14 to 28), which is consistent with clinical guidelines on ‘good practice’ for length of prescriptions in the UK.^4^

Using these data, we estimated that a prescription date is likely to fall at any point in the 60 days before the date of reimbursement (based on the time-lag described above) and be fully dispensed by 12 days before the date of reimbursement (based on an average of 40 days between prescription and reimbursement, with a prescription length of 28 days (as informed by available data, above)). We therefore coded new treatment episodes as commencing 60 days before the first date of reimbursement and ending 12 days before the last date of reimbursement. We defined a continuous episode of treatment as a pattern of regular monthly dates of reimbursement with no more than two months between them. As prescription data may be incomplete in longer treatment episodes and prescriptions may span two months (as described above), we hypothesized that a shorter period (i.e., one missed monthly reimbursement) may not be indicative of a gap in treatment. All remaining follow up time was coded as 'off' treatment.

We included in our study individuals who received at least one prescription between 2011 and 2020. We used prescriptions from 2010 to identify those who were already exposed to treatment at the start of follow-up on 1 January 2011; for those without a prescription in 2010, the start of follow-up was defined as the first estimated date of prescription.

### **Outcome: Mortality data**

Date and cause of death were obtained from Scotland’s death registry, maintained by National Records Scotland. All death records are CHI-identified. The primary outcome measure for this study was Suicide (including undetermined intent).

Suicides were identified based on the ICD-10 codes reported as the underlying and/or contributing cause of death (Table 1). This definition follows the ‘baseline’ set of ICD-10 codes used by National Records Scotland.^5^

**Table 1: Description and ICD-10 codes of outcomes and covariates.**

| **Description** | | **ICD-10 Codes** |
| --- | --- | --- |
| *Suicide* | |  |
|  | Intentional self-harm by drugs | X60, X61, X62, X63, X64 |
|  | Intentional self-harm by other means | X65, X66, X67, X68, X69, X70, X71, X72, X73, X74, X75, X76, X77, X78, X79, X80, X81, X82, X83, X84 |
|  | Undetermined intent  Sequelae of intentional self-harm  Sequelae of events of undetermined intent | Y10, Y11, Y12, Y13, Y14, Y15, Y16, Y17, Y18, Y19, Y20, Y21, Y22, Y23, Y24, Y25, Y26, Y27, Y28, Y29, Y30, Y31, Y32, Y33, Y34  Y87·0  Y87·2 |
| *Admission for mental and behavioural disorder* | |  |
|  | Schizophrenia and psychoses | F20, F21, F22, F23, F24, F25, F26, F27, F28, F29 |
|  | Mood [affective] disorders | F30, F31, F32, F33, F39, F34·0, F34·1, F34·8, F34·9, F43·21, F43·23 |
|  | Neurotic, stress-related and somatoform disorders | F40, F41, F42, F43 (excluding F43·21, F43·23), F44, F45, F46, F47, F48 |
|  | Disorders of adult personality and behaviour | F60, F61, F62, F63, F64, F65, F66, F67, F68, F69 |
|  | Attention deficit hyperactivity disorder | F90 |
| *Admission for self-harm* | |  |
|  | Intentional self-harm by drugs  Intentional self-harm by other means | X60, X61, X62, X63, X64  X65, X66, X67, X68, X69, X70, X71, X72, X73, X74, X75, X76, X77, X78, X79, X80, X81, X82, X83, X84 |

**References**

1. McAuley A, Fraser R, Glancy M, et. al. Mortality among individuals prescribed opioid-agonist therapy in Scotland, UK, 2011-20: a national retrospective cohort study. *Lancet Public Health* 2023; **8:** e484-e493.

2. Alvarez-Madrazo S, McTaggart S, Nangle C, Nicholson E, Bennie M. Data Resource Profile: The Scottish National Prescribing Information System (PIS). *Int J Epidemiol* 2016; **45:** 714-715f.

3. Gao L, Dimitropoulou P, Robertson JR, McTaggart S, Bennie M, Bird SM. Risk-factors for methadone-specific deaths in Scotland's methadone-prescription clients between 2009 and 2013. *Drug Alcohol Depend* 2016; **167:** 214‐223.

4. Clinical Guidelines on Drug Misuse and Dependence Update 2017 Independent Expert Working Group. Drug misuse and dependence: UK guidelines on clinical management. 2017. <https://assets.publishing.service.gov.uk/government/uploads/system/uploads/attachment_data/file/673978/clinical_guidelines_2017.pdf>

5. National Records of Scotland (NRS). Probable Suicides 2022. September 5, 2023. [Probable suicides 2022, Report (nrscotland.gov.uk)](https://www.nrscotland.gov.uk/files/statistics/probable-suicides/2022/suicides-22-report.pdf) (accessed Nov 12, 2023).

#

# **Supplementary File S2**

## **INCLUSION AND EXCLUSION CRITERIA**

**Figure 1: Flowchart describing cohort inclusion and exclusion criteria.**


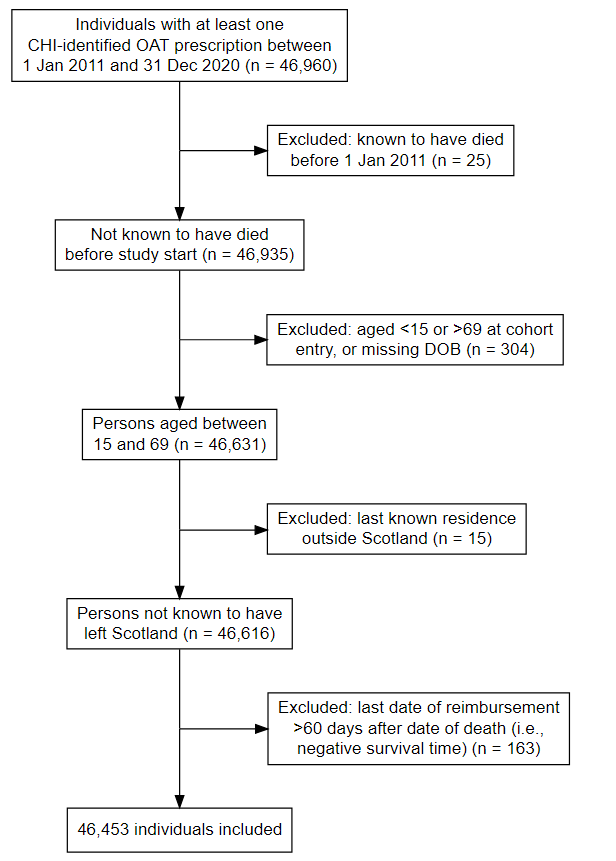


# **Supplementary File S3**

## **FURTHER DETAILS ON COHORT CHARACTERISTICS**

**Table 1: Further Characteristics of the study cohort, first reported in McAuley et al 2023^1^**

| **Variable** | **N (%)*** |
| --- | --- |
| **Total** | 46 453 |
| **Health board of residence (last known) (missing = 563, 1·1 %)** |  |
| Greater Glasgow & Clyde | 14 153 (30·5%) |
| Tayside | 3 873 (8·3%) |
| Other | 27 864 (60·0%) |
| **Mental health disorder hospital admission (at cohort-entry)** |  |
| No | 41 658 (89·7%) |
| Yes | 4 795 (10·3%) |
| **Prescriber type (at cohort-entry)** |  |
| GP | 28 365 (61·1%) |
| Other | 18 088 (38·9%) |
| **Year of first OAT prescription within study period** |  |
| 2010 | 26 356 (56·7%) |
| 2011-2012 | 5 698 (12·3%) |
| 2013-2014 | 4 403 (9·5%) |
| 2015-2016 | 3 846 (8·3%) |
| 2017-2018 | 3 222 (7·2%) |
| 2019-2020 | 2 828 (6·1%) |
| **Year of last OAT prescription within study period** |  |
| 2011-2012 | 2 865 (6·2%) |
| 2013-2014 | 3 066 (6·6%) |
| 2015-2016 | 3 545 (7·6%) |
| 2017-2018 | 4 450 (9·6%) |
| 2019-2020 | 32 527 (70·0%) |
| **OAT-type (at cohort-entry)** |  |
| Methadone | 39 255 (84·5%) |
| Buprenorphine | 2 373 (5·1%) |
| Buprenorphine/Naloxone | 4 825 (10·4%) |
| **Ever prescribed** |  |
| Buprenorphine only | 1 627 (3·5%) |
| Methadone only | 31 682 (68·2%) |
| Buprenorphine/Naloxone only | 1 596 (3·4%) |
| Methadone and Buprenorphine and/or Buprenorphine/Naloxone | 10 154 (21·9%) |
| Buprenorphine and Buprenorphine/Naloxone | 1 384 (3·0%) |
| **Last prescribed** |  |
| Buprenorphine | 5 499 (11·8%) |
| Methadone | 37 584 (80·9%) |
| Buprenorphine/Naloxone | 3 370 (7·3%) |
| **Total time on OAT across all episodes, years** |  |
| Median (IQR) | 4·72 years (1·80 to 7·74) |
| Range | 1 day to 10 years |
| **Number of distinct treatment periods during follow-up** |  |
| Median (IQR) | 3 (1 to 5) |
| Range | 1 to 23 |
| **Length of treatment periods** |  |
| Median (IQR) | 201 days (79 to 566) |
| Range | 1 day to 10 years |

*unless otherwise specified.

**Reference**

1. McAuley A, Fraser R, Glancy M, et. al. Mortality among individuals prescribed opioid-agonist therapy in Scotland, UK, 2011-20: a national retrospective cohort study. *Lancet Public Health* 2023; **8:** e484-e493.

# **SUPPLEMENTARY FILE S4**

## **SENSIVITY ANALYSIS**

### **(i) EXCLUDING SUICIDES OF UNDETERMINED INTENT**

**Table 1: Crude Suicide Mortality Rates, Hazard Ratios and Adjusted Hazard Ratios for Suicides among people prescribed OAT in Scotland, 2011-2020: excluding suicides of undetermined intent.**

| **Variable** | **Deaths** | **Person Years** | **Mortality / 1,000 PY (95% CI)** | **Hazard Ratio*** | **P-value** | **Adj. Hazard Ratio*** | **P-value** |
| --- | --- | --- | --- | --- | --- | --- | --- |
| **Total** | 334 | 304 043 | 1·10 (0·99, 1·22) |  |  |  |  |
| **OAT exposure** |  |  |  |  |  |  |  |
| On | 145 | 223 973 | 0·65 (0·55, 0·76) | 1 (ref) |  | 1 (ref) |  |
| Off | 189 | 80 070 | 2·36 (2·05, 2·72) | 3·15 (2·54, 3·90) | <0·001 | 3·14 (2·52, 3·90) | <0·001 |
| **Sex** |  |  |  |  |  |  |  |
| Male | 255 | 203 776 | 1·25 (1·11, 1·41) | 1 (ref) |  | 1 (ref) |  |
| Female | 79 | 100 267 | 0·79 (0·63, 0·98) | 0·64 (0·49, 0·82) | <0·001 | 0·61 (0·47, 0·79) | <0·001 |
| **Age** |  |  |  |  |  |  |  |
| Under 35 | 94 | 81 989 | 1·15 (0·94, 1·40) | 1·01 (0·77, 1·31) | 0·967 | 0·93 (0·71, 1·22) | 0·589 |
| 35 – 44 | 152 | 139 043 | 1·09 (0·93, 1·28) | 1 (ref) |  | 1 (ref) |  |
| 45 – 54 | 88 | 83 011 | 1·06 (0·86, 1·31) | 0·94 (0·72, 1·22) | 0·641 | 1·02 (0·78, 1·34) | 0·877 |
| **Time Period** |  |  |  |  |  |  |  |
| 2011-2012 | 69 | 57 890 | 1·19 (0·94, 1·51) | 1 (ref) |  | 1 (ref) |  |
| 2013-2014 | 70 | 60 670 | 1·15 (0·91, 1·46) | 1·17 (0·77, 1·78) | 0·455 | 1·12 (0·75, 1·66) | 0·578 |
| 2015-2016 | 54 | 61 720 | 0·87 (0·67, 1·14) | 0·90 (0·57, 1·43) | 0·668 | 0·82 (0·53, 1·26) | 0·362 |
| 2017-2018 | 73 | 62 310 | 1·17 (0·93, 1·47) | 1·32 (0·87, 2·00) | 0·188 | 1·18 (0·80, 1·75) | 0·412 |
| 2019-2020 | 68 | 61 460 | 1·11 (0·87, 1·40) | 1·30 (0·87, 1·96) | 0·204 | 1·10 (0·75, 1·63) | 0·626 |
| **SIMD (deprivation index)** |  |  |  |  |  |  |  |
| SIMD 3, 4, 5 (least deprived) | 53 | 60 522 | 0·88 (0․67, 1·15) | 1 (ref) |  | 1 (ref) |  |
| SIMD 2 | 88 | 76 014 | 1·16 (0·94, 1·43) | 1·34 (0·95, 1·88) | 0·095 | 1·39 (0·99, 1·95) | 0·060 |
| SIMD 1 (most deprived) | 193 | 167 507 | 1·15 (1·00, 1·33) | 1·33 (0·98, 1·80) | 0·068 | 1·45 (1·07, 1·96) | 0·018 |
| **Mental Health Disorder Admission** |  |  |  |  |  |  |  |
| No | 263 | 274 146 | 0·96 (0·85, 1·08) | 1 (ref) |  | 1 (ref) |  |
| Yes | 71 | 29 897 | 2·37 (1·88, 3·00) | 2·40 (1·84, 3·11) | <0·001 | 2·14 (1·61, 2·84) | <0·001 |
| **Self-harm Hospital Admission** |  |  |  |  |  |  |  |
| No | 208 | 234 132 | 0·89 (0·78, 1·02) | 1 (ref) |  | 1 (ref) |  |
| Yes | 126 | 69 911 | 1·80 (1·51, 2·15) | 1·95 (1·56, 2·43) | <0·001 | 1·80 (1·42, 2·28) | <0·001 |
| **Prescriber Type** |  |  |  |  |  |  |  |
| General Practitioner | 216 | 194 621 | 1·11 (0·97, 1·27) | 1 (ref) |  | 1 (ref) |  |
| Other | 118 | 109 422 | 1·08 (0·90, 1·29) | 0·96 (0·77, 1·21) | 0·756 | 0·82 (0·65, 1·03) | 0·095 |
| **Charlson Comorbidity Index** |  |  |  |  |  |  |  |
| 0 (none) | 243 | 195 481 | 1·24 (1·10, 1·41) | 1 (ref) |  | 1 (ref) |  |
| 1-2 (medium) | 69 | 74 173 | 0·93 (0·73, 1·18) | 0·74 (0·56, 0·96) | 0·024 | 0·67 (0·51, 0·88) | 0·005 |
| 3+ (high) | 22 | 34 388 | 0·64 (0·42, 0·97) | 0·46 (0·30, 0·71) | <0·001 | 0·39 (0·24, 0·61) | <0·001 |

*Modelled using competing risks regression, with all-cause mortality (excluding suicides) as the competing event.

### **(ii) REMOVING CENSORING OF FOLLOW UP TIME**

**Table 2: Crude Suicide Mortality Rates, Hazard Ratios and Adjusted Hazard Ratios for Suicides among people prescribed OAT in Scotland, 2011-2020, removing censoring of follow-up.**

| **Variable** | **Deaths** | **Person Years** | **Mortality / 1,000 PY (95% CI)** | **Hazard Ratio*** | **P-value** | **Adj. Hazard Ratio*** | **P-value** |
| --- | --- | --- | --- | --- | --- | --- | --- |
| **Total** | 629 | 347 745 | 1·81 (1·67, 1·96) |  |  |  |  |
| **OAT exposure** |  |  |  |  |  |  |  |
| On | 256 | 224 327 | 1·14 (1·01, 1·29) | 1 (ref) |  | 1 (ref) |  |
| Off | 373 | 123 419 | 3·02 (3·73, 3·35) | 2·53 (2·16, 2·97) | <0·001 | 2·57 (2·18, 3·02) | <0·001 |
| **Sex** |  |  |  |  |  |  |  |
| Male | 453 | 231 852 | 1·95 (1·78, 2·14) | 1 (ref) |  | 1 (ref) |  |
| Female | 176 | 115 894 | 1·52 (1·31, 1·76) | 0·79 (0·66, 0·94) | 0·007 | 0·77 (0·65, 0·92) | 0·004 |
| **Age** |  |  |  |  |  |  |  |
| Under 35 | 155 | 90 681 | 1·71 (1·46, 2·00) | 0·84 (0·69, 1·03) | 0·094 | 0·79 (0·65, 0·97) | 0·027 |
| 35 – 44 | 290 | 158 285 | 1·83 (1·63, 2·06) | 1 (ref) |  | 1 (ref) |  |
| 45 – 54 | 184 | 98 780 | 1·86 (1·61, 2·15) | 1·03 (0·85, 1·24) | 0·788 | 1·09 (0·90, 1·32) | 0·374 |
| **Time Period** |  |  |  |  |  |  |  |
| 2011-2012 | 149 | 58 041 | 2·57 (2·19, 3·01) | 1 (ref) |  | 1 (ref) |  |
| 2013-2014 | 132 | 65 906 | 2·00 (1·69, 2·38) | 0·84 (0·62, 1·13) | 0·244 | 0·82 (0·62, 1·09) | 0·169 |
| 2015-2016 | 112 | 71 398 | 1·57 (1·30, 1·89) | 0·73 (0·53, 0·99) | 0·042 | 0·67 (0·50, 0·90) | 0·008 |
| 2017-2018 | 125 | 75 114 | 1·66 (1·40, 1·98) | 0·79 (0·58, 1·07) | 0·129 | 0·71 (0·53, 0·96) | 0·024 |
| 2019-2020 | 111 | 77 287 | 1·44 (1·19, 1·73) | 0·73 (0·54, 0·99) | 0·042 | 0·62 (0·46, 0·84) | 0·002 |
| **SIMD (deprivation index)** |  |  |  |  |  |  |  |
| SIMD 3, 4, 5 (least deprived) | 121 | 72 043 | 1·68 (1·41, 2·01) | 1 (ref) |  | 1 (ref) |  |
| SIMD 2 | 171 | 87 133 | 1·96 (1·69, 2·28) | 1·18 (0·93, 1·49) | 0·167 | 1·21 (0·96, 1·53) | 0·103 |
| SIMD 1 (most deprived) | 337 | 188 569 | 1·79 (1·61, 1·99) | 1·07 (0·87, 1·32) | 0·514 | 1·14 (0·92, 1·40) | 0·230 |
| **Mental Health Disorder Admission** |  |  |  |  |  |  |  |
| No | 505 | 313 658 | 1·61 (1·48, 1·76) | 1 (ref) |  | 1 (ref) |  |
| Yes | 124 | 34 088 | 3·64 (3·05, 4·34) | 2·18 (1·79, 2·65) | <0·001 | 1·96 (1·59, 2·43) | <0·001 |
| **Self-harm Hospital Admission** |  |  |  |  |  |  |  |
| No | 419 | 268 938 | 1·56 (1·42, 1·71) | 1 (ref) |  | 1 (ref) |  |
| Yes | 210 | 78 808 | 2·66 (2·33, 3·05) | 1·63 (1·38, 1·93) | <0·001 | 1·51 (1·27, 1·81) | <0·001 |
| **Prescriber Type** |  |  |  |  |  |  |  |
| General Practitioner | 402 | 222 346 | 1·87 (1·69, 2·07) | 1 (ref) |  | 1 (ref) |  |
| Other | 227 | 125 400 | 1·93 (1·68, 2·21) | 0·99 (0·84, 1·16) | 0·869 | 0·92 (0·78, 1·08) | 0·313 |
| **Charlson Comorbidity Index** |  |  |  |  |  |  |  |
| 0 (none) | 439 | 226 789 | 1·94 (1·77, 2·13) | 1 (ref) |  | 1 (ref) |  |
| 1-2 (medium) | 130 | 82 867 | 1·57 (1·32, 1·86) | 0·80 (0·65, 0·97) | 0·011 | 0·73 (0·59, 0·89) | 0·002 |
| 3+ (high) | 60 | 38 090 | 1·58 (1·22, 2·03) | 0·72 (0·55, 0·94) | 0·015 | 0·59 (0·44, 0·79) | <0·001 |

*Modelled using competing risks regression, with all-cause mortality (excluding suicides) as the competing event.

### **(iii) COVID-19 CENSORING**

**Table 3: Crude Suicide Mortality Rates, Hazard Ratios and Adjusted Hazard Ratios for Suicides among people prescribed OAT in Scotland, 2011-2020, restricting follow-up to end on 29^th^ February 2020.**

| **Variable** | **Deaths** | **Person Years** | **Mortality / 1,000 PY (95% CI)** | **Hazard Ratio*** | **P-value** | **Adj. Hazard Ratio*** | **P-value** |
| --- | --- | --- | --- | --- | --- | --- | --- |
| **Total** | 531 | 278 551 | 1·91 (1·75, 2·08) |  |  |  |  |
| **OAT exposure** |  |  |  |  |  |  |  |
| On | 237 | 206 263 | 1·15 (1·01, 1·31) | 1 (ref) |  | 1 (ref) |  |
| Off | 294 | 72 287 | 4·07 (3·63, 4·56) | 3·10 (2·61, 3·68) | <0·001 | 3·15 (2·65, 3·75) | <0·001 |
| **Sex** |  |  |  |  |  |  |  |
| Male | 382 | 186 567 | 2·05 (1·85, 2·26) | 1 (ref) |  | 1 (ref) |  |
| Female | 149 | 91 984 | 1·62 (1·38, 1·90) | 0·80 (0·66, 0·97) | 0·020 | 0·80 (0·66, 0·97) | 0·026 |
| **Age** |  |  |  |  |  |  |  |
| Under 35 | 135 | 77 816 | 1·74 (1·47, 2·05) | 0·83 (0·67, 1·03) | 0·083 | 0·75 (0·60, 0·94) | 0·012 |
| 35 – 44 | 246 | 128 172 | 1·92 (1·69, 2·17) | 1 (ref) |  | 1 (ref) |  |
| 45 – 54 | 150 | 72 573 | 2·07 (1·76, 2·43) | 1·08 (0·88, 1·32) | 0·481 | 1·20 (0·97, 1·47) | 0·094 |
| **Time Period** |  |  |  |  |  |  |  |
| 2011-2012 | 149 | 57 890 | 2·57 (2·19, 3·02) | 1 (ref) |  | 1 (ref) |  |
| 2013-2014 | 123 | 60 670 | 2·03 (1·70, 2·42) | 0·82 (0·60, 1·12) | 0·201 | 0·79 (0·59, 1·05) | 0·107 |
| 2015-2016 | 99 | 61 721 | 1·60 (1·32, 1·95) | 0·72 (0·52, 1·00) | 0·053 | 0·65 (0·48, 0·89) | 0·006 |
| 2017-2018 | 113 | 62 310 | 1·81 (1·51, 2·18) | 0·80 (0·58, 1·11) | 0·189 | 0·73 (0·54, 0·99) | 0·046 |
| 2019-2020 | 47 | 35 964 | 1·31 (0·98, 1·74) | 0·55 (0·38, 0·79) | 0·001 | 0·50 (0·36, 0·70) | <0·001 |
| **SIMD (deprivation index)** |  |  |  |  |  |  |  |
| SIMD 3, 4, 5 (least deprived) | 92 | 55 214 | 2·09 (1·36, 2·04) | 1 (ref) |  | 1 (ref) |  |
| SIMD 2 | 145 | 69 484 | 2·09 (1·77, 2·46) | 1·27 (0·98, 1·65) | 0·074 | 1·30 (1·00, 1·70) | 0·046 |
| SIMD 1 (most deprived) | 294 | 153 852 | 1·91 (1·70, 2·14) | 1·17 (0·92, 1·47) | 0·199 | 1·23 (0·97, 1·56) | 0·080 |
| **Mental Health Disorder Admission** |  |  |  |  |  |  |  |
| No | 420 | 251 081 | 1·67 (1·52, 1·84) | 1 (ref) |  | 1 (ref) |  |
| Yes | 111 | 27 469 | 4·04 (3·35, 4·87) | 2·34 (1·90, 2·89) | <0·001 | 2·08 (1·66, 2·62) | <0·001 |
| **Self-harm Hospital Admission** |  |  |  |  |  |  |  |
| No | 345 | 214 641 | 1·61 (1·45, 1·79) | 1 (ref) |  | 1 (ref) |  |
| Yes | 186 | 63 910 | 2·91 (2·52, 3·36) | 1·74 (1·46, 2·08) | <0·001 | 1·60 (1·32, 1·95) | <0·001 |
| **Prescriber Type** |  |  |  |  |  |  |  |
| General Practitioner | 342 | 179 343 | 1·91 (1·72, 2·12) | 1 (ref) |  | 1 (ref) |  |
| Other | 189 | 99 207 | 1·91 (1·65, 2·20) | 0·98 (0·82, 1·18) | 0·859 | 0·88 (0·74, 1·06) | 0·186 |
| **Charlson Comorbidity Index** |  |  |  |  |  |  |  |
| 0 (none) | 381 | 178 158 | 2·14 (1·93, 2·36) | 1 (ref) |  | 1 (ref) |  |
| 1-2 (medium) | 105 | 68 238 | 1·54 (1·27, 1·86) | 0·71 (0·57, 0·88) | 0·002 | 0·62 (0·50, 0·78) | <0·001 |
| 3+ (high) | 45 | 32 154 | 1·40 (1·04, 1·87) | 0·59 (0·43, 0·81) | 0·001 | 0·45 (0·32, 0·62) | <0·001 |

*Modelled using competing risks regression, with all-cause mortality (excluding suicides) as the competing event.

### **(iv) DRUG RELATED DEATHS (EXCLUDING SUICIDES) AS THE COMPETING EVENT**

**Table 4****: Crude Suicide Mortality Rates, Hazard Ratios and Adjusted Hazard Ratios for Suicides among people prescribed OAT in Scotland, 2011-2020, with Drug-related Deaths (excluding suicides) defined as the competing event.**

| **Variable** | **Deaths** | **Person Years** | **Mortality / 1,000 PY (95% CI)** | **Hazard Ratio*** | **P-value** | **Adj Hazard Ratio*** | **P-value** |
| --- | --- | --- | --- | --- | --- | --- | --- |
| **Total** | 575 | 304 043 | 1·89 (1·74, 2·05) |  |  |  |  |
| **OAT exposure** |  |  |  |  |  |  |  |
| On | 256 | 223 973 | 1·14 (1·01, 1·29) | 1 (ref) |  | 1 (ref) |  |
| Off | 319 | 80 070 | 3·98 (3·57, 4·45) | 3·30 (2·81, 3·89) | <0·001 | 3·24 (2·74 to 3·83) | <0·001 |
| **Sex** |  |  |  |  |  |  |  |
| Male | 415 | 203 776 | 2·04 (1·85, 2·24) | 1 (ref) |  | 1 (ref) |  |
| Female | 160 | 100 267 | 1·60 (1·37, 1·86) | 0·78 (0·65, 0·94) | 0·009 | 0·79 (0·65 to 0·95) | 0·012 |
| **Age** |  |  |  |  |  |  |  |
| Under 35 | 148 | 81 989 | 1·81 (1·54, 2·12) | 0·88 (0·72, 1·09) | 0·238 | 0·79 (0·64, 0·98) | 0·029 |
| 35 – 44 | 265 | 139 043 | 1·91 (1·69, 2·15) | 1 (ref) |  | 1 (ref) |  |
| 45 – 54 | 162 | 83 011 | 1·95 (1·67, 2·28) | 1·08 (0·89, 1·32) | 0·424 | 1·18 (0·96, 1·45) | 0·107 |
| **Time Period** |  |  |  |  |  |  |  |
| 2011-2012 | 149 | 57 890 | 2·57 (2·19, 3·02) | 1 (ref) |  | 1 (ref) |  |
| 2013-2014 | 123 | 60 670 | 2·03 (1·70, 2·42) | 0·76 (0·54, 1·07) | 0·116 | 0·73 (0·53, 1·00) | 0·052 |
| 2015-2016 | 99 | 61 720 | 1·60 (1·32, 1·95) | 0·65 (0·46, 0·93) | 0·019 | 0·58 (0·42, 0·81) | 0·001 |
| 2017-2018 | 113 | 62 310 | 1·81 (1·51, 2·18) | 0·73 (0·51, 1·04) | 0·080 | 0·65 (0·46, 0·90) | 0·010 |
| 2019-2020 | 91 | 61 460 | 1·48 (1·21, 1·82) | 0·61 (0·43, 0·88) | 0·007 | 0·52 (0·37, 0·72) | <0·001 |
| **SIMD (deprivation index)** |  |  |  |  |  |  |  |
| SIMD 3, 4, 5 (least deprived) | 105 | 60 522 | 1·73 (1·43, 2·10) | 1 (ref) |  | 1 (ref) |  |
| SIMD 2 | 157 | 76 014 | 2·07 (1·77, 2·42) | 1·20 (0·94, 1·54) | 0·151 | 1·23 (0·96, 1·57) | 0·107 |
| SIMD 1 (most deprived) | 313 | 167 507 | 1·87 (1·67, 2·09) | 1·09 (0·87, 1·36) | 0·462 | 1·14 (0·91, 1·42) | 0·258 |
| **Mental Health Disorder Admission** |  |  |  |  |  |  |  |
| No | 458 | 274 146 | 1·67 (1·52, 1·83) | 1 (ref) |  | 1 (ref) |  |
| Yes | 117 | 29 897 | 3·91 (3·26, 4·69) | 2·29 (1·87, 2·81) | <0·001 | 2·01 (1·61, 2·50) | <0·001 |
| **Self-harm Hospital Admission** |  |  |  |  |  |  |  |
| No | 375 | 234 132 | 1·60 (1·45, 1·77) | 1 (ref) |  | 1 (ref) |  |
| Yes | 200 | 69 911 | 2·86 (2·49, 3·29) | 1·76 (1·48, 2·09) | <0·001 | 1·68 (1·31, 1·91) | <0·001 |
| **Prescriber Type** |  |  |  |  |  |  |  |
| General Practitioner | 364 | 194 621 | 1·87 (1·69, 2·07) | 1 (ref) |  | 1 (ref) |  |
| Other | 211 | 109 422 | 1·93 (1·68, 2·21) | 1·06 (0·89, 1·25) | 0·527 | 0·91 (0·77, 1·09) | 0·309 |
| **Charlson Comorbidity Index** |  |  |  |  |  |  |  |
| 0 (none) | 409 | 195 481 | 2·09 (1·90, 2·31) | 1 (ref) |  | 1 (ref) |  |
| 1-2 (medium) | 115 | 74 173 | 1·55 (1·29, 1·86) | 0·73 (0·59, 0·90) | 0·003 | 0·66 (0·54, 0·82) | <0·001 |
| 3+ (high) | 51 | 34 388 | 1·48 (1·13, 1·95) | 0·68 (0·51, 0·91) | 0·010 | 0·59 (0·44, 0·81) | 0·001 |

*Modelled using competing-risks regression, with drug-related deaths (excluding suicides) as the competing event.

# **SUPPLEMENTARY FILE S5**

## **ASSOCIATIONS BETWEEN SUICIDE, OAT, SEX AND AGE GROUP**

**Figure 1: Crude Suicide Mortality Rates over time for males, by OAT exposure.**

**Figure 2: Crude Suicide Mortality Rates over time for females, by OAT exposure.**

**Figure 3: Crude Suicide Mortality Rates over time for males, by age group.**

# **SUPPLEMENTARY FILE S6**

## **STANDARDISED MORTALITY RATIO (SMR) BREAKDOWN AND COMPARISON**

**Table 1: Age & Sex Standardised Mortality Ratio (SMR) calculation breakdown.**

|  |  | **OAT cohort** | | | **NRS^1^** | |
| --- | --- | --- | --- | --- | --- | --- |
| **Sex** | **Age Groups** | **Observed no· suicides** | **Person Years** | **Mortality Rate/1,000 PY** | **Age-specific suicide rates per 1,000 (2011-2020)*** | **Expected no· suicides** |
| **Male** | 15-19 | 0 | 161 | 0 | 0·12 | 0·02 |
|  | 20-24 | 6 | 2 643 | 2·27 (1·02, 5·05) | 0·23 | 0·60 |
|  | 25-29 | 27 | 12 756 | 2·12 (1·45, 3·09) | 0·29 | 3·67 |
|  | 30-34 | 69 | 31 757 | 2·17 (1·72, 2·75) | 0.32 | 10.19 |
|  | 35-39 | 89 | 47 756 | 1·86 (1·51, 2·29) | 0.37 | 17.53 |
|  | 40-44 | 100 | 47 607 | 2·10 (1·73, 2·56) | 0.38 | 17.90 |
|  | 45-49 | 73 | 34 469 | 2·12 (1·48, 2·84) | 0.37 | 12.68 |
|  | 50-54 | 36 | 17 594 | 2·05 (1·48, 2·84) | 0.31 | 5.45 |
|  | 55-59 | 11 | 6 320 | 1·74 (0·96, 3·14) | 0.27 | 1.69 |
|  | 60-64 | 4 | 2 012 | 1·99 (0·75, 5·30) | 0.20 | 0.39 |
|  | 65-69 | 0 | 703 | 0 | 0.15 | 0.11 |
| **Female** | 15-19 | 0 | 215 | 0 | 0·05 | 0·01 |
|  | 20-24 | 5 | 3 051 | 1·64 (0·68, 3·94) | 0·07 | 0·22 |
|  | 25-29 | 19 | 10 806 | 1·76 (1·12, 2·76) | 0·08 | 0·88 |
|  | 30-34 | 22 | 20 600 | 1·07 (0·70, 1·62) | 0.09 | 1.92 |
|  | 35-39 | 40 | 24 183 | 1·65 (1·21, 2·26) | 0.13 | 3.07 |
|  | 40-44 | 36 | 19 498 | 1·85 (1·33, 2·56) | 0.13 | 2.50 |
|  | 45-49 | 19 | 12 480 | 1·52 (0·97, 2·39) | 0.13 | 1.67 |
|  | 50-54 | 13 | 6 156 | 2·11 (1·23, 3·64) | 0.12 | 0.76 |
|  | 55-59 | 5 | 2 245 | 2·23 (0·93, 5·35) | 0.10 | 0.22 |
|  | 60-64 | 1 | 759 | 1·32 (0·19, 9·36) | 0.08 | 0.06 |
|  | 65-69 | 0 | 273 | 0 | 0.06 | 0.02 |
| **Total** | | **575** |  |  |  | **81·55** |
| SMR = (Observed no· suicides/expected no· suicides) | | 575/81·55 = 7**·05** | | | | |
| 95% Confidence intervals | | (6·50, 7·65) | | | | |

*Standardised using Scotland’s age-specific mortality rates stratified by gender for years 2011-2020^1^

**Table 2: Comparison of SMRs to other studies**

|  | **Observed no. suicides** | **Expected no. suicides** | **SMR** | **95% CI** | **England SMR^2^ (1998-2017)** | **Australia SMR^3^ (2002-2017)** |
| --- | --- | --- | --- | --- | --- | --- |
| **Age SMR** | 575 | 68·99 | 8·34 | (7·68-9·05) | N/A | N/A |
| **Age & Sex SMR** | 575 | 81·55 | 7·05 | (6·50, 7·65) | 7·51 (5·50 – 10·02) | 5·6 (3·62, 7·66) |

**References**

1. National Records of Scotland (NRS). Probable Suicides 2022. September 5, 2023. [Probable suicides 2022, Report (nrscotland.gov.uk)](https://www.nrscotland.gov.uk/files/statistics/probable-suicides/2022/suicides-22-report.pdf) (accessed Nov 12, 2023).
2. Padmanathan P, Forbes H, Redaniel MT, et. al. Self-harm and suicide during and after opioid agonist treatment among primary care patients in England: a cohort study. *Lancet Psychiatry* 2022; **9:** 151-159.
3. Colledge-Frisby S, Jones N, Degenhardt L, et. al. Incidence of suicide and self-harm among people with opioid use disorder and the impact of opioid agonist treatment: A retrospective data linkage study. *Drug Alcohol Dependence* 2023; **1:** 109851.

# **SUPPLEMENTARY FILE S7**

## **ATTRIBUTABLE RISK FRACTION (ARF) CALCULATION**

ARF = *P*_1_(RR-1)/1+*P*_1_(RR-1)^1^

Where:

P1 = proportion (prevalence) of those exposed to drugs in the population. **1·32%^2^**

RR = sex and age adjusted standardised mortality ratio (SMR) for suicides in the OAT cohort. **7·05**

= 0·0132(7·05-1)/1+0·0132(7·05-1) = 0·15972 = **15·97% (95% CI: 14·52%, 17·56%)**

**References**

1. Levin ML. Symposium on endemiology of cancer of lung: Occurrence of lung cancer in man. *Acta Unio Int Contra Cancrum* 1953; **9:** 531-41.
2. Markoulidakis A, Hickman M, McAuley A, et al. Prevalence of opioid dependence in Scotland 2015-2020: a Multi-Parameter Estimation of Prevalence (MPEP) Study. *Addiction*. 2024; 119(8):1410-1420.
